# Ovotesticular Disorder With Seminoma

**DOI:** 10.7759/cureus.12130

**Published:** 2020-12-17

**Authors:** Subha R Samantray, Ipsita Mohapatra

**Affiliations:** 1 Obstetrics and Gynecology, Prathima Institute of Medical Sciences, Karimnagar, IND

**Keywords:** ovotesticular disorder, disorders of sex development, true hermaphroditism, seminoma, orchiectomy

## Abstract

Ovotesticular disorder or true hermaphroditism is defined as the presence of both ovarian and testicular tissues in the same individual irrespective of the patient’s karyotype. Ovotesticular disorder represents 5% of disorders of sex development. Cases of true hermaphroditism must be treated like men or women based on their age, external genitalia and the orientation of the patient. Here we report a case of true hermaphroditism which was diagnosed with seminoma on histopathological examination. The patient underwent orchiectomy, followed by two cycles of chemotherapy.

## Introduction

Ovotesticular disorder or true hermaphroditism is defined as the presence of both ovarian and testicular tissues in the same individual irrespective of the patient’s karyotype. It is an extremely rare condition with a prevalence rate of less than 1/20,000 [1]. About 60% of the patients have 46XX karyotype, 33% have 46XX/46XY sex chromosome mosaicism, while 7% have 46 XY karyotype [2]. Ovotesticular disorder represents 5% of disorders of sex development (DSD) [3]. The risk of acquiring adverse gonadal and genitourinary pathologies increases in patients with ovotestis. They may present with gonadoblastomas, hypospadias, cryptorchidism, other types of aberrant anatomy or rarely with seminoma. Seminoma is a low-grade testicular tumour arising from the germ cells. Here we report a case of true hermaphroditism which was diagnosed with seminoma on histopathological examination.

## Case presentation

A 20-year-old young man presented with severe lower abdominal pain since four days. He complained of dull aching pain in the left iliac region for the last month, which was intensified for the last four days. The pain was not associated with fever, burning micturition or vomiting. The pain was relieved on taking antispasmodics and on taking rest. The patient did not complain of any specific discomfort in his past medical history was also unremarkable. There was no blood relationship between his parents, and his elder brother was a healthy normal male with two children. On examination, patient was phenotypically a normal male with well developed external male genitalia. He had a normal male distribution of hair. The external phallus was normal in size, and urethral orifice was present in normal position with no hypospadias. On palpation, the right testes were present in the right scrotal sac and were normal in size. However, the left scrotum was empty. There was no lymphadenopathy. Patient described of having erections but had no experience of sexual activity. In the left iliac region, tenderness was elicited, but no mass was palpable.

Following admission, investigations revealed normal levels of total Testosterone (482.82ng/dl) and Estradiol (16pg/ml) for a male. Luteinizing hormone (16.32mIU/ml) and follicle-stimulating hormone (28.74mIU/L) were elevated. All these findings are consistent with primary testicular failure. Serum Prolactin and Dehydroepiandrosterone levels as well as the thyroid function tests, were normal. The cytogenetic analysis of the karyotype showed genotype 46XY.

Abdominopelvic ultrasonography showed a well-circumscribed, ovoid, heterogeneously hypoechoic lesion of 2.2×2.6×1.3 cm size in the left iliac region. It also showed the presence of a small uterus adjacent to the bladder wall. MRI showed multinodular, sharply defined, 2.1×2.6×1.2cm homogeneous tumour of low signal intensity on T2-weighted images and areas of signal intensity heterogeneity related to haemorrhage or necrosis. MRI findings suggested seminomatous changes of the left testis.

**Figure 1:**
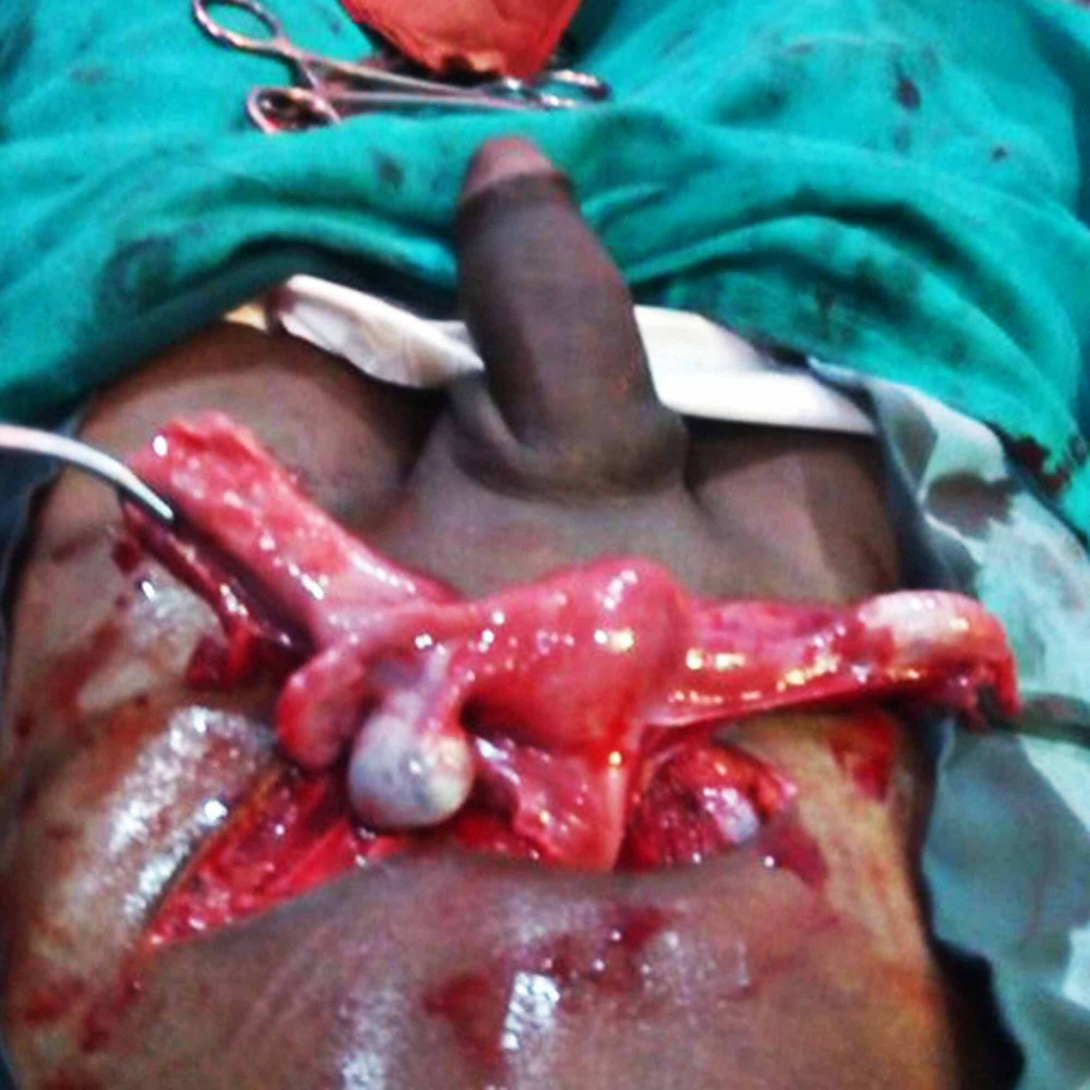
Intra-operative picture showing small uterus with bilateral tubes and ovaries, phallus

**Figure 2:**
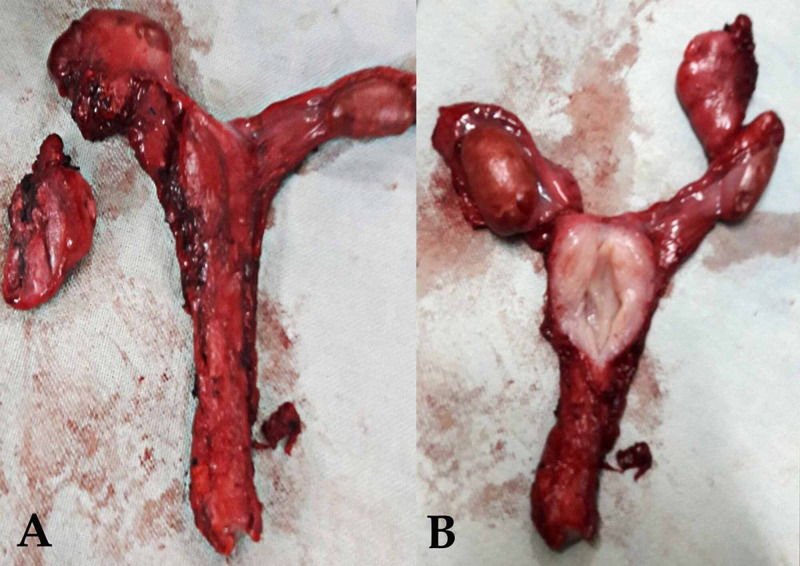
Cut section of surgical specimen of uterus showing normal endometrium and cavity

Patient and his relatives were correctly counselled about the condition. Efforts were made to counsel the patient psychologically. After proper surgical workup, an exploratory laparotomy was planned. There were a small, well-developed uterus, fallopian tubes and bilateral ovaries (Figure 1). As the patient had been reared as a male and given consent for removal of the uterus, total abdominal hysterectomy with bilateral salpingo-oophorectomy was done. Uterus, fallopian tubes and ovaries were normal on a cut section (Figure 2). Cervix could not be delineated anatomically, and there was no vagina present.

**Figure 3:**
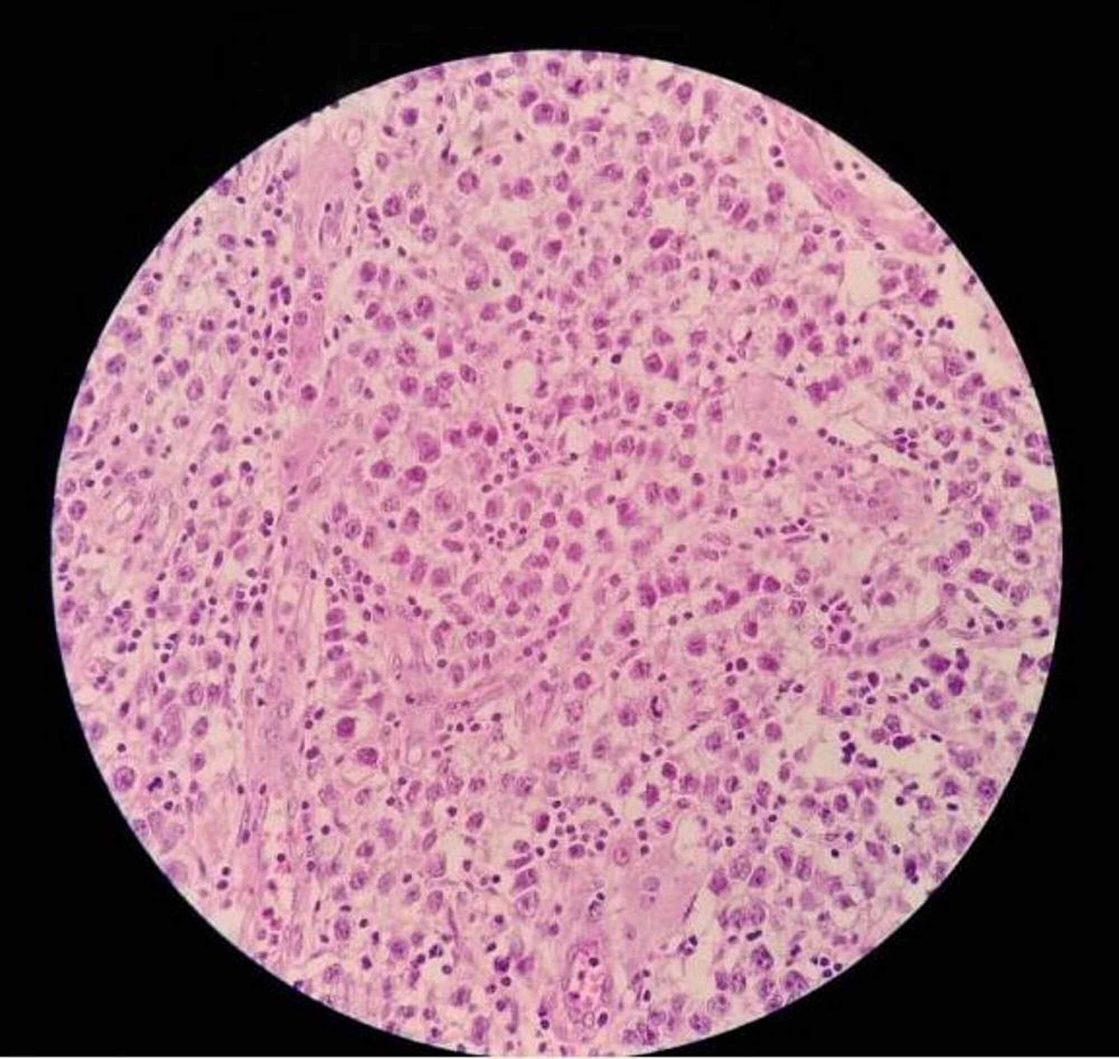
Histopathological section shows tumour cells arranged in sheets of uniform cells in monotonous population, divided into poorly demarcated lobules by delicate fibrous tissue septa and sparse collection of lymphocytes in between the cells suggestive of testicular seminoma

The left testis was present near the left deep inguinal ring. It was rubbery in consistency and the surface looked normal. There was no enlargement of the local lymph nodes. The left testis was removed in toto because of the high chances of malignancy suggested by MRI. A biopsy was taken from the right testicle by making a small incision over the scrotal skin in order to rule out any dysgenesis or ovotesticular tissue. Right testicular biopsy showed normal histology with seminiferous tubules without any dysgenesis or ovarian tissue. The histology of the ovarian sections showed ovarian parenchymal tissue with primary and secondary follicles, a corpus luteum, and several corpora albicantia. Histopathological examination revealed seminoma of the left testis (Figure 3).

Patient was discharged on 7th postoperative day. The patient was referred to the oncologist for further management. Two cycles of chemotherapy were planned and the patient was counselled for sperm banking before chemotherapy. He could not go for sperm banking due to financial constraints. Postoperatively two cycles of chemotherapy with carboplatin were given. Patient is in regular follow up with the oncologist and is having no complaints at this time.

## Discussion

True hermaphroditism diagnosis is made based on the histological presence of both testicular and ovarian tissue in the same individual. Almost 90% of subjects will present at birth with ambiguous genitalia like microphallus, hypospadias, urogenital sinus, a fusion of penoscrotal labia, or cryptorchidism [3]. Ovotesticular disorder of sex development is the rarest form of DSD accounting for about 5%. The exact cause of this condition is not known. However, abnormalities of sex chromosome, abnormal gonadal development and endocrine abnormalities during embryonic development may have a role in the pathogenesis of Ovotesticular disorder [4]. SRY gene acts as the testes determining factor. Any mutation in this gene can also result in Ovotesticular disorder. Exposure to exogenous sex hormones like Oestrogen or Progesterone during pregnancy can also lead to abnormalities of sexual development [5]. The gonads may be present in any of the combination of the ovary, testes or combined ovary and testes. Presence of ovotestis is most common, followed by the presence of ovaries. The presence of testes is least common. The position of ovotestis depends mainly on the amount of testicular tissue present [6]. Based on the location of gonads and histology, these patients are classified as 1. Lateral: Testis and contralateral ovary (30%), 2. Bilateral: Testicular and ovarian tissue identified on both sides, usually as ovotestis (50 %), 3. Unilateral: Ovotestis on one side and testis or ovary on another side (20%) [7]. Our patient had a presence of testes and ovaries on both sides, which were well developed (bilateral category).

The karyotype of patients can be 46XX (60%), 46 XY(7%), other mosaic karyotypes like 46XX/46XY, 46XY/45X(33%) [2]. Two-third of real hermaphroditism cases live as males [8]. In cases where the androgen secretion is not sufficient during the embryonic stage, the scrotum and penis are not developed at birth, and hence patient may lead a life as a woman. The urethral orifice may be present on the underside of the penis. An undescended testis is one of the most common presentations. In our case, patient had well developed external genitalia and secondary sexual characters and was leading a normal male life.

Radiological examination plays a vital role in the assessment of DSD cases. For diagnosis, sex hormone testing, ultrasound, computed tomography and magnetic resonance imaging can be done. Ultrasound remains the modality of choice in the evaluation of patients with ambiguous genitalia as there is no exposure to radiation or contrast. In some cases, endoscopy or diagnostic laparoscopy is needed in addition to radiological imaging [9]. However, surgical exploration and histopathological confirmation of the presence of both type of gonads is the gold standard for confirmation of a diagnosis of Ovotesticular disorder [8]. In our case, both ovaries were present. Right testes were present in the right scrotal sac while the left testes had not descended and were lying near the right deep inguinal ring.

True hermaphroditism is rarely associated with gonadal tumours. Some cases of malignancies like dysgerminoma and gonadoblastoma have been reported in true hermaphroditism subjects [10]. As the malignancy rate is very low, so prophylactic removal of gonads is not indicated. Germ cell tumours are most common with dysgerminoma being the most common histological variant [3]. Seminoma in patients of Ovotesticular disorders is extremely rare. Seminoma is primordial germ cell tumour of the testes. They account for a third of all testicular germ cell tumours with a survival rate of 98-99% if treated in early stages [11]. They are low-grade tumours occurring in middle-aged men. Seminoma is common in cases of crypto-orchidism due to more temperature in the abdominal cavity [12]. Metastasis may occur through lymphatic or haematogenous route. Placental alkaline phosphatase, alpha-fetoprotein (AFP), lactate dehydrogenase (LDH), human chorionic gonadotropin (β-hCG) and CD117 are tumour markers used for the diagnosis of seminoma [11]. Testicular seminoma is very sensitive to radiation therapy and chemotherapy. Surgery combined with chemo or radiotherapy can significantly improve the prognosis. Our case was diagnosed with stage 1 seminoma of left testes based on surgical staging and given two cycles of chemotherapy with carboplatin after orchiectomy of left testes.

Cases of true hermaphroditism must be treated like men or women based on their age, external genitalia and the orientation of the patient. Surgical treatment followed by hormonal support is the mainstay of treatment. In cases where the gonads are associated with malignant changes, it should be removed with postoperative chemotherapy or radiotherapy given according to the type and stage of the disease. To prevent malignancy, corrective surgery is recommended for undescended testes in children before the age of two years.

## Conclusions

While most of the Ovotesticular disorder cases present with the ambiguous genitalia during infancy, some cases may also come into light during adulthood as seen in our case. A multidisciplinary team should do the management of a patient with Ovotesticular disorder. DSD management may include medical treatment, surgical correction of ambiguous genitalia and removal of dysgenic gonads or Mullerian components, and psychological counselling.
